# Proteomic Analysis and Label-Free Quantification of the Large *Clostridium difficile* Toxins

**DOI:** 10.1155/2013/293782

**Published:** 2013-08-27

**Authors:** Hercules Moura, Rebecca R. Terilli, Adrian R. Woolfitt, Yulanda M. Williamson, Glauber Wagner, Thomas A. Blake, Maria I. Solano, John R. Barr

**Affiliations:** ^1^Division of Laboratory Sciences, National Center for Environmental Health, Centers for Disease Control and Prevention (CDC), MS F-50, 4770 Buford Hwy NE, Atlanta, GA 30341, USA; ^2^Association of Public Health Laboratories, Silver Spring, MD 20910, and Oak Ridge Institute for Scientific Education, Oak Ridge, TN 37380, USA; ^3^Universidade do Oeste de Santa Catarina, 89600 Joacaba, SC, Brazil

## Abstract

*Clostridium difficile* is the leading cause of antibiotic-associated diarrhea in hospitals worldwide, due to hypervirulent epidemic strains with the ability to produce increased quantities of the large toxins TcdA and TcdB. Unfortunately, accurate quantification of TcdA and TcdB from different toxinotypes using small samples has not yet been reported. In the present study, we quantify *C. difficile* toxins in <0.1 mL of culture filtrate by quantitative label-free mass spectrometry (MS) using data-independent analysis (MS^E^). In addition, analyses of both purified TcdA and TcdB as well as a standard culture filtrate were performed using gel-based and gel-independent proteomic platforms. Gel-based proteomic analysis was then used to generate basic information on toxin integrity and provided sequence confirmation. Gel-independent in-solution digestion of both toxins using five different proteolytic enzymes with MS analysis generated broad amino acid sequence coverage (91% for TcdA and 95% for TcdB). Proteomic analysis of a culture filtrate identified a total of 101 proteins, among them TcdA, TcdB, and S-layer proteins.

## 1. Introduction

*Clostridium difficile* is a gram-positive, anaerobic, spore-forming, rod-shaped bacterium that can produce at least three toxins including two Rho GTPase-glucosylating toxins (TcdA and TcdB) and the binary *C. difficile* transferase (CDT) toxin. The organism can cause *C. difficile* infection (CDI) in humans and animals. *C. difficile* is considered an important cause of healthcare-associated infection in humans and is the leading cause of antibiotic-associated diarrhea in hospitals worldwide [1, 2]. CDIs are toxin-mediated illnesses that range from mild diarrhea to fulminant pseudomembranous colitis and toxic megacolon, which may result in death [3]. Laboratorial confirmation of CDI needs to be rapidly performed. The methods include detection of *C. difficile* through cultivation, detection of Tcd A and Tcd B in stool samples using immunoassay and DNA-based methods [2].

Reflecting its changing epidemiology, the incidence and severity of CDI have increased significantly in the past ten years. Among the possible causes of increasing morbidity is the emergence of strains considered to be more virulent [4]. These strains produce greater amounts of toxin than reference strains and are highly transmissible due to their greater sporulation capacity [4]. One example is the rapid emergence of the highly virulent clone-designated BI/NAP1/027 in multiple countries [5]. Additionally, it is estimated that there are ~500,000 cases of CDI per year in US hospitals and long-term facilities alone, with an estimated ~15,000 to 30,000 deaths [2]. Since the illness may be severe and is difficult to treat and there is currently no available vaccine, preventing individual cases and outbreaks of CDI is a major challenge. 

Although it has been demonstrated [4, 5] that strains or toxinotypes associated with outbreaks and high morbidity produce more toxin than historic, nonepidemic isolates, the expression levels of these toxins from different toxinotypes are not completely known. Moreover, the toxin load in clinical samples and culture supernatants has always been roughly estimated [5–8], and the accurate quantification of TcdA and TcdB using small samples has not yet been reported. Accurate quantification of toxins can be accomplished using proteomic strategies which may reveal key information applicable to TcdA and TcdB method development, detection, understanding toxic mechanisms, and production of new therapeutics including polyclonal and monoclonal antibodies [2].

Proteomics has been described as a key technology in the postgenomic era that provides information complementary to that provided by genomics. Proteins can be analyzed rapidly, accurately, and with high sensitivity using mass spectrometry (MS). Proteomic examinations have been performed for *C. difficile* in which proteins released *in vitro* during high toxin production [7] were identified for strains 630 and VPI 10461. Additional studies characterizing the subproteomes of *C. difficile* reference strain 630, including a surface protein and insoluble protein fraction analysis [9, 10], spore protein identification [11], and a protein assessment associated with heat stress responses, have also been reported [12]. In an additional study, culture supernatants of *C. difficile* reference strain 630 were compared to two hypervirulent strains (CD196 and CDR20291), and five secreted proteins were identified exclusively in the supernatants of the hypervirulent strains [13]. 

Absolute protein quantification by MS is a well-studied technique typically performed using stable isotope dilution [14–16]. However, applying a data-independent analysis (MS^E^) that does not require labeled compounds and is amenable to sample-limited experiments [17] is a newly available alternative. In MS^E^, data are acquired in a data-independent fashion using an alternating low/high-energy scan mass analysis and can be used to perform both protein identification and quantification in one MS experiment [18, 19]. 

We have previously described MS-based proteomics studies of different bacterial toxins [20], including botulinum neurotoxin [21, 22], anthrax lethal factor [20], and pertussis toxin [23]. In the present study, we describe a gel-based proteomic analysis of TcdA and TcdB. A gel-independent approach was also used in which the toxins were digested by five different proteolytic enzymes to maximize amino acid sequence coverage. Toxin digests were analyzed qualitatively using two different MS instrument platforms and were further analyzed using a label-free quantitative methodology. This study provides the performance characteristics and the basis for future development of improved MS-based detection and quantification methods for TcdA and TcdB and may help to identify protein factors involved in *C. difficile* toxin production by different isolates. We expect that such efforts will contribute to a better understanding of these toxin-mediated illnesses and will lead to new preventive measures and therapies against *C. difficile* infection. 

## 2. Materials and Methods

### 2.1. *Clostridium difficile* Toxins

**Figure 1:**
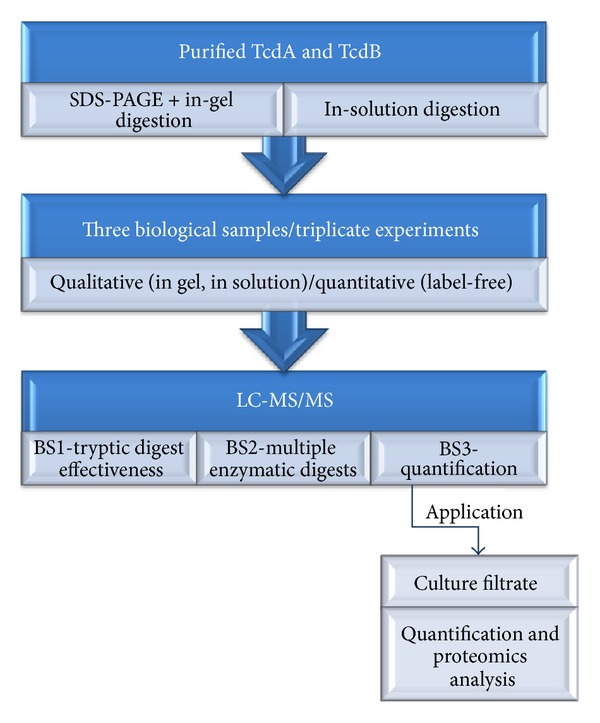
Schematic flow diagram of baseline data and biomarker discovery methods to study the large *C. difficile* toxins.

Purified TcdA (one lot—lot 1-15215A1C) and TcdB (two lots—lot 1-1551A1B and lot 2-15518A1B) used in this study were purchased from List Biologicals Laboratories Inc. (Campbell, CA, USA). The toxins were purified from *C. difficile* VPI 10463—ATCC 43255 and were provided in vials containing 100 *μ*g of TcdA or 20 *μ*g of TcdB lyophilized in 0.05 M Tris. Three vials of each toxin (TcdA lot 1, TcdB lot 2) were assigned numbers in house and designated as distinct biological samples (BioS1, BioS2, and BioS3). The vials containing TcdA and TcdB were reconstituted as per the manufacturer's instructions, aliquoted, and used immediately as the designated BioS. Aliquots (20 *μ*L) from the first biological sample (BioS1) were used to run SDS-PAGE gels and for three separated fast trypsin in-solution digestions to verify method reproducibility [24]. Ten microliters aliquots from samples designated as BioS2 were digested using five different enzymes in parallel to obtain maximum protein sequence coverage. Aliquots of BioS3 were serially diluted (1 : 2 factor, starting at 2 *μ*g and ending at 0.125 *μ*g) and used to determine the sensitivity of the MS^E^ method used in this study. In addition, a commercially available lyophilized *C. difficile* culture filtrate (CFil) control reagent (Techlab *Clostridium difficile* Toxin/Antitoxin Kit—T5000, Blacksburg, VA, USA) was used for both the quantitative method and qualitative proteomic analyses. A schematic flow diagram of the procedures used in this work can be found in Figure 1. All chemicals used in this study were purchased from Sigma-Aldrich (St.Louis, MO, USA) unless otherwise indicated.

### 2.2. SDS-PAGE Analysis and In-Gel Digestion

Duplicates of purified TcdA lot 1 (BioS1) and two lots of TcdB lot 1 and lot 2 (BioS1) were treated with NuPAGE (Invitrogen Carlsbad, CA, USA) sample buffer and the proteins were separated by 1D SDS-PAGE using the NuPAGE Novex system (Bis-tris-gels; 4–12% polyacrylamide gradient) as per the manufacturer's instructions (Invitrogen). In addition, BioS1 in-solution tryptic digests were separated using Tricine gels (Invitrogen). The gels were then either stained with Colloidal Coomassie Blue (GelCode Blue Safe Protein Stain—Thermo Scientific, Pierce, Rockford, IL, USA) or with Pierce Silver Stain Kit for Mass Spectrometry (Thermo Scientific). After being scanned, each gel band was cut into slices of approximately 0.4 cm. In-gel digestion was performed with sequence grade trypsin (Promega, Madison, WI, USA) as previously described [21]. Briefly, the gel slices were dried for 30 min using a Centrivap concentrator (Labconco, Kansas City, MO, USA) and 10 *μ*L of trypsin (0.5 *μ*g/*μ*L) diluted in 25 *μ*L of a 50 mM ammonium bicarbonate solution, pH 8.5, containing 1 mM calcium chloride (digestion buffer), was added to each sample. After 5 min incubation at room temperature (RT), 25 *μ*L of digestion buffer was added and the samples were incubated at 37°C overnight (ON). Following ON incubation, the digests were quenched with 0.1% formic acid (FA), sonicated for 3 min, and centrifuged at 1200 g for 10 min. The supernatants were used for nanoscale ultrapressure liquid chromatography (nUPLC)-MS/MS analysis. 

### 2.3. In-Solution Enzymatic Digestion

In-solution detergent-based 3 min tryptic digestions were performed as described [21], using three aliquots of purified TcdA (lot1) and TcdB (lot2) (BioS1, S2, and S3) (1 *μ*g/*μ*L) and CFil (10 *μ*g/*μ*L). Ten microliters of 0.2% RapiGest (RG), an acid-labile surfactant (Waters Corporation, Milford, MA, USA), in-digestion buffer, was added to each 10 *μ*L-aliquot of TcdA, TcdB, and CFil and the tubes were incubated at 99°C for 5 min using a thermocycler (Applied Biosystems, Foster City, CA, USA). The solution was rapidly cooled to RT, and trypsin (~50 pmol) in-digestion buffer, was added. The samples were incubated at 52°C for 3 min. To hydrolyze the RG, 10 *μ*L of 0.45 M HCl was added and the samples were incubated at 37°C for 30 min. The suspension was further diluted to a final volume of 50 *μ*L with 0.1% FA and centrifuged (1200 g) for 10 min. The supernatant containing the peptides was frozen at −70°C if not used immediately. For calibration of the quantification method, yeast alcohol dehydrogenase (ADH) standard tryptic digest solution (Waters Corporation) was added to the supernatant before analysis by MS^E^, at a concentration required to give 100 fMol ADH on column. Each toxin was digested in triplicate (technical replicates) and submitted to the MS instrument in triplicates (analytical replicates) and the results of all MS runs were compared. To visually evaluate digestion effectiveness, BioS1 tryptic digests were analyzed using SDS-PAGE tricine gels and silver stained. Four additional enzymes, AspN, chymotrypsin, GluC, and LysC (Sigma-Aldrich, San Louis, MO, USA), were separately used to digest aliquots of BioS2 in order to maximize proteome coverage. Enzymatic digestions were performed as per the manufacturer's instructions.

### 2.4. MS Analyses and Database Search

Liquid chromatography-tandem mass spectrometry (LC-MS/MS) was carried out using an nUPLC coupled either to a QTof Premier MS system (Waters Corporation, Milford, MA, USA), or to a linear ion trap (LTQ)-Velos Orbitrap tandem MS instrument (Thermo Scientific, San Jose, CA, USA). All the conditions for nUPLC separation, (including flow rate and solvent concentrations) as well as the MS^E^ method on the QTof Premier MS, were used as previously described [21]. 

Each digest was analyzed in triplicate (three analytical replicates per digest sample), and the respective raw data files obtained using data-independent LC-MS^E^ were further processed using the ProteinLynx Global Server v2.4 software (PLGS, Waters Corporation), for protein identification and quantification. Database searches were performed using the PLGS Identity^E^ database search algorithm against either a UniProt protein database (November 2009; 6 × 10^5^ entries) or a modified NCBInr database created with the term “difficile” (December 2010; 3.5 × 10^3^ entries) to which the ADH 30,030 Da amino acid sequence was added. The PLGS software package provided statistically validated peptide and protein identification along with the determination of the stoichiometry of the protein constituents of the mixture (relative quantification) along with the expected amounts of protein present in the mixture (absolute quantification). The remaining parameters, including mass accuracy for precursor (10 ppm) and product (20 ppm) ions and criteria for protein identifications, were defined as before [21]. Similarly, relative protein quantification for the ADH digest-spiked samples (100 fMol on the column) was obtained using both the PLGS Identity^E^ and the Expression software [18, 19]. The clustered dataset was exported from PLGS and further analyzed with Microsoft Excel 2010 (Microsoft Corporation, Redmond, WA, USA). Scaffold (v3.01, Proteome Software Inc., Portland, OR, USA) was used to further validate MS/MS based peptide and protein identifications as before [21]. The reported data represent three technical replicates and three analytical replicates.

Additionally, protein digests were analyzed using an LTQ Velos Orbitrap tandem MS instrument. Peptides were separated using an nUPLC system directly coupled online to the MS instrument through an Advance Captive Spray source from Michrom Bioresources (Auburn, CA, USA). The spray voltage was set at 1500 V, and the capillary temperature was 200°C. nUPLC separation was performed as previously described [21]. Briefly, the mobile phase consisted of (solvent A) 0.2% FA, 0.005% trifluoroacetic acid (TFA) in water, and (solvent B) 0.2% FA, 0.005% TFA in ACN. The gradient was set at 5% B for 5 min, followed by a ramp to 40% B over 90 min, and then a ramp up to 95% B in 1 min. The gradient was then held at 95% B for 5 min before returning to 5% B in 2 min, followed by reequilibration at 5% B for 5 min. 

The MS was programmed to perform data-dependent acquisition by scanning the mass range from *m/z* 400 to 1400 at a nominal resolution setting of 60,000 FMHM for parent ion acquisition in the Orbitrap. Tandem mass spectra of doubly charged and higher charge state ions were acquired for the top 15 most intense ions in each survey scan. All tandem mass spectra were recorded by use of the linear ion trap. This process cycled continuously throughout the duration of the nUPLC gradient. All tandem mass spectra were extracted from the raw data file using Mascot Distiller (Matrix Science, London, UK; version 2.2.1.0) and searched using Mascot (version 2.2.0). Mascot was setup to search using the entire NCBInr database or a modified NCBInr database created to search “*C. difficile*” recognized proteins, or to search for *C. difficile* strain ATCC 43255 in which trypsin is used as the digestion agent. Mascot and Scaffold search parameters were used as described before with stringent parameters so the probability of a wrong assignment was below 0.1% [22, 23]. PSORTb subcellular scores were used to predict and localize identified culture supernatant proteins (http://www.psort.org/psortb/) [24]. NCBI gi accession numbers were employed to assign functions to each of the identified proteins using KEGG identifiers http://www.genome.jp/kegg/kegg3.html as described before [23].

## 3. Results

### 3.1. Gel-Based Proteomics Platform

**Figure 2:**
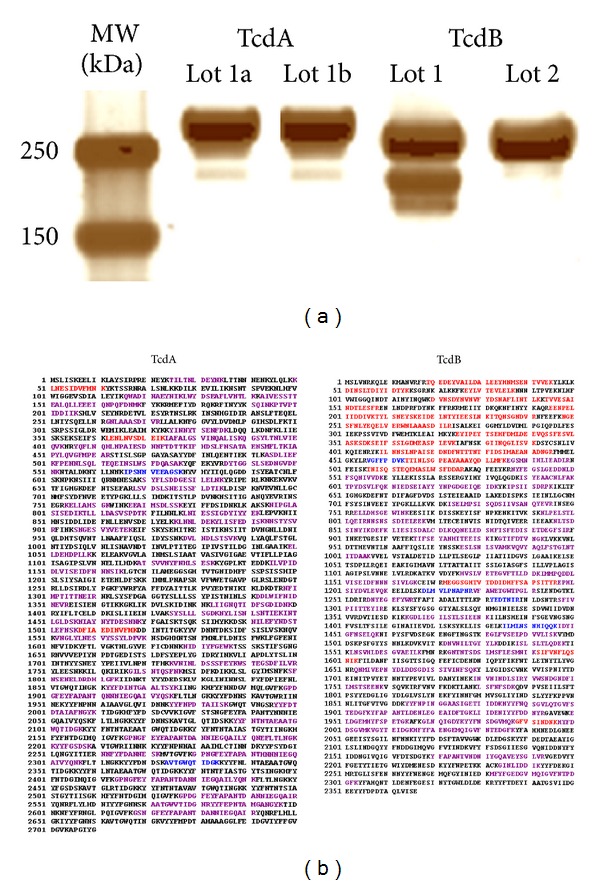
Gel-based analysis of purified *C. difficile* toxins. (a) SDS-PAGE of purified TcdA and TcdB. Only one band was observed in the TcdA lanes; two bands were observed in the TcdB lot 1 lane; one band was observed in the TcdB lot 2 lane as expected. (b) Amino acid sequence coverage obtained for the two lots of *C. difficile* toxins. The gel bands were extracted, digested, and MS analyzed. Peptide sequences detected were overlaid. Sequences in Red = peptides from run 1a (TcdA) or band 1 (TcdB); Blue = peptides from run 1b or band 2; Purple = common peptides; Black = not detected.

1D SDS-PAGE followed by silver stain revealed intense bands corresponding to purified TcdA and TcdB proteins (Figure 2(a)). Interestingly, in the lane containing TcdB (lot 1), an extra, prominent band was present at the ~210 kDa MW region. However, analysis of TcdB (lot 2) revealed only one band at ~270 kDa, as expected. Trypsin digestion and MS analysis of all the protein-extracted gel bands confirmed their amino acid (AA) sequences as TcdA and TcdB. It also indicated that the extra-band in lot 1 represents a truncated TcdB, in which peptides corresponding to AA 1 through 543 are missing. This finding can be observed in Figure 2(b) through the analysis of the matched peptides detected after overlaying the amino acid sequences detected in the two excised gel bands from lot 1.

### 3.2. Gel-Independent Proteomics Platform

First, three separate aliquots of BioS1 (TcdA lot1 and TcdB lot2) were used to verify the effectiveness of the 3 min digestion method for the large *C. difficile* toxins. As revealed by nUPLC-MS/MS analysis, in-solution digestion of the toxins generated a large peptide pool. Additionally, to confirm completeness of digestion, analysis of the digests using a silver-stained Tricine gel (not shown) revealed no bands, suggesting completeness of digestion.

Secondly, BioS2 aliquots were digested in parallel using multiple enzymes. The amino acid percent coverage, for TcdA and TcdB for each enzyme used, was 37% and 34% for AspN, 28% and 35% for chymotrypsin, 40% and 61% for GluC, 8% and 57% for LysC, and 80% and 66% for trypsin. The two enzymes that delivered the most complementary sequence coverage were trypsin and GluC (combined sequence coverage of 83% for TcdA and 86% for TcdB). Summation of the entire MS peptide analysis revealed broad amino acid sequence coverage for TcdA (91%) and TcdB (95%).

Finally, BioS3 was used for the absolute quantification of TcdA and TcdB in small samples using MS^E^ (Table 1). The minimum amounts of digested TcdA and TcdB in buffer that can be loaded on the nUPLC column and still be detected were, respectively, 5 ng (1.6 *μ*g/mL) and 1.25 ng (0.43 *μ*g/mL). 

**Figure 3:**
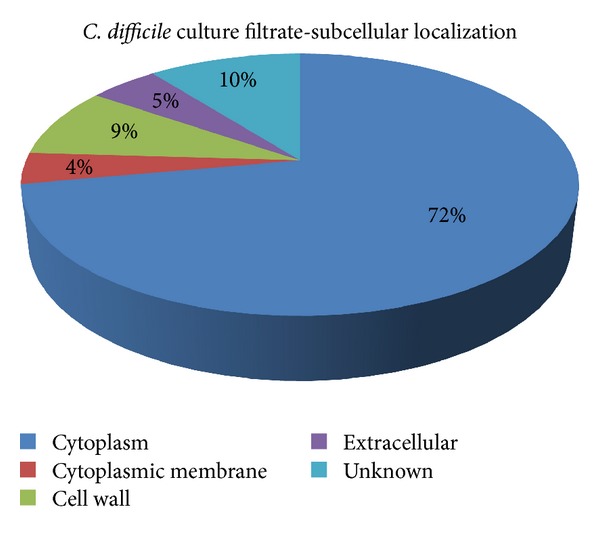
Subcellular localization of *C. difficile* proteins identified in a commercial culture filtrate using Psort score. Most proteins were cytoplasmatic (72%).

A further application of the MS^E^ method was to successfully determine the relative and absolute amounts of TcdA and TcdB in a commercial lyophilized *C. difficile* culture filtrate (CFil) control reagent. In addition, because MS^E^ data analysis can be used to determine the relative abundance of proteins in a complex mixture, data obtained with the CFil were processed and 24 constituents of the filtrate could be quantified (Table 2). Data analysis clearly shows that the S-layer proteins were the most abundant proteins in the mixture, followed by TcdA and TcdB. After proteomic analysis of the CFil using both MS instruments, a total of 101 proteins were identified. With the stringent parameters used for Peptide Prophet and Protein Prophet within the Scaffold software, the false discovery rate was zero. Psort and KEGG localization disclosed that most proteins (72%) were cytoplasmatic (Figure 3).

## 4. Discussion

The large toxins TcdA and TcdB are the major virulence factors of *Clostridium difficile* and are primary markers for diagnosis of CDI. These toxins are glycosyltransferases involved in the inactivation of small GTPases which is a key factor in disease pathogenesis. The present proteomic study is foundational and is aimed at exploring the potential of this methodology for the development of a mass spectrometry-based method for accurate TcdA and TcdB quantification. Gel-based 1D SDS-PAGE analysis of TcdA and TcdB was performed, followed by in-gel trypsin digestion of the proteins and further mass spectrometric analysis. A gel-independent approach was also performed using in-solution multienzymatic digestion of TcdA and TcdB followed by nUPLC-MS/MS, with both data-dependent analysis and LC-MS^E^. Data-independent analysis is advantageous as it can potentially provide both absolute and relative label-free quantification results. 

The gel-based approach provided critical information on TcdA and TcdB protein integrity and amino acid (AA) sequence coverage maps. The expected unique protein band (~300 kDa MW) was visible in the TcdA lane, while two bands were observed in the TcdB lane for the first lot of a commercially available toxin. Further MS analysis of the gel-excised tryptic-digested proteins from this first lot demonstrated that the band at the ~270 kDa region was the complete TcdB, whereas the extra-band (~210 kDa) was a truncated TcdB protein missing the N-terminal peptides (~68 kDa) which are associated with enzymatic activity. Interestingly, the presence of two bands at ~210 and ~68 kDa is normally expected when TcdB is autocleaved by the cysteine-protease present in the toxin molecule during the activation process which can occur *in vivo* or *in vitro* [1, 2]. However, in this case, it is possible that self-cleavage did occur and the 68 kDa portion was lost during the manufacturer's purification process since no band for the N-terminal fragment was observed. Fortunately, analysis of a second lot of TcdB from the same vendor only revealed the expected ~270 kDa band, and this lot was used in all further experiments. 

The gel-independent approach provided unique information related to toxin quantification of small samples (<0.1 mL). The first set of experiments using BioS1 confirmed the robustness and efficiency of our modified rapid trypsin digestion method [21]. While trypsin provided sufficient AA sequence coverage to unambiguously identify TcdA and TcdB, using one enzyme may not provide enough AA sequence information to differentiate toxins produced by different *C. difficile* strains. The use of multiple proteases has been reported before not only to improve AA coverage, but also to differentiate homologous proteins, detect posttranslational modifications, and identify editing events [25–27]. For initial discovery work, five different enzymes were used to obtain broad *C. difficile* AA coverage. However, since high-quality proteases are expensive, the use of multiple enzymes for each sample can be cost prohibitive. Working towards this, careful analysis of TcdA and TcdB AA sequence coverage maps from all five enzymes proved that using only two enzymes, trypsin and GluC, collectively garnered enough sequence information for most of our applications.

Quantification of TcdA and TcdB in samples irrespective of the volume capacity has typically been performed using enzyme-linked immunosorbent assays (ELISA) [4–8], cytotoxity assays [8, 28, 29], PCR [30], and gel densitometry [6]. These methods are limited in that they generally measure total toxin and do not discriminate between TcdA and TcdB.

We report here the use of data-independent nUPLC-MS^E^ to separately quantify *C. difficile* toxins in small sample volumes. After assessing the MS^E^ spectrum data, standard curves were constructed for TcdA and TcdB, and the minimum amounts detected were determined for each toxin. 

A review of MS^E^ concepts and applications has been published by others [18, 19]. In addition, a recent systematic evaluation of the MS^E^ method has been reported. The authors found that there is a linear dynamic range of three orders of magnitude and low limit of quantification when they tested complex mixtures in small volumes [31]. Since the nUPLC-MS^E^ methodology is expected to be very sensitive [31], the obtained values reported in the present work for both toxins may not be ideal for samples containing small amounts of toxin. Therefore, our current research focuses on improving the sensitivity of our nUPLC-MS^E^ method, coupling it with other known toxin concentration methods such as antibody capture prior to MS analysis [20]. 

Previously, we have used nUPLC-MS^E^ for the quantification of botulinum toxin complexes [21, 22]. These findings taken with the successful quantification of purified *C. difficile* large toxins reported here led us to examine a commercial culture filtrate (CFil) to determine nUPLC-MS^E^ utility for analysis of a complex *C. difficile* mixture. A proteomic analysis of CFil, normally used as a positive control in toxicity assays, revealed the identification of 101 unique *C. difficile* proteins, of which 24 could be quantified by MS^E^. The proteins identified are comparable with results from previous *C. difficile* proteomic studies [13]. Most importantly, proteomic quantitative analysis of a culture filtrate demonstrated the potential of these methods for further studies.

Interestingly, label-free quantification of CFil identified four proteins (S-layer protein, TcdA, TcdB, and NAD-specific glutamate dehydrogenase) all detected in significantly large amounts. One possible explanation is that these proteins are traditionally abundant and thus would likely be detected in greater amounts. In addition, they are large proteins, which once digested likely have a larger peptide pool compared to other less abundant proteins, resulting in a greater chance for detection by nUPLC-MS^E^. Even more, other proteins, such as glycine cleavage system protein H, isocaprenoyl-CoA:2-hydroxyisocaproate CoA-transferase, and molecular chaperone DnaK, that have not been previously cited in proteomic studies were also detected in lower but significant amounts. This finding suggests that culture supernatants may harbor a pool of specific proteins and could be a key matrix to search for potential unique *C. difficile* biomarkers. Because of the high TcdA and TcdB toxin concentration detected in the CFil, we initially hypothesized that the filtrate had been enriched by the manufacturer. However, we were assured by the manufacturer that the CFil used in these studies is indeed a filtrate of the culture supernatant of the strain VPI 10463, ATCC43255, which has not undergone any type of enhancement treatment, besides filtration, that would concentrate the protein pool present in this matrix (personal communication). Interestingly, a recent publication describes the proteome examination of culture supernatants of three *C. difficile* strains, two of them hypervirulent isolates [13]. The authors used gel-based analysis followed by MS and identified 5 unique proteins among the hypervirulent strains. They emphasized the usefulness of proteomics to discover and quantify specific biomarkers for hypervirulent strains since at least 234 unique genes have been identified in the strains that cause the majority of hospital outbreaks in North America and Europe [32].

## 5. Conclusions

Proteomic analyses using gel-based and gel-independent platforms were used to further characterize and quantitate the large *C. difficile* toxins. The overall goal was to determine the potential of proteomics using mass spectrometry-based methods to develop a quantification method for these large toxins. Moreover, the use of MS^E^ for *C. difficile* toxin quantification in small samples, and for multienzymatic digestion to increase protein amino acid sequence coverage, was performed. The gel-based work revealed basic information on toxin integrity and provided sequence confirmation. The gel-independent platform was applied to in-solution digestion of both toxins using five different enzymes followed by analysis using two different mass spectrometer instruments. Broad amino acid sequence coverage for TcdA (91%) and for TcdB (95%) was generated using this approach. These data, if coupled to *in silico* sequencing analysis, suggest that the method has the potential to determine subtle sequence differences of TcdA and TcdB from different *C. difficile* toxinotypes. Moreover, label-free proteomics using MS^E^ data collection and analysis provided the ability potential to determine the absolute quantity of TcdA and TcdB in small samples and was applied to a culture filtrate. A proteomic study of the culture filtrate demonstrated that the most abundant proteins are S-layer protein, TcdA, TcdB, and NAD-specific glutamate dehydrogenase. Taken together, data presented in this study provide performance characteristics and the basis for future development of improved MS-based detection and quantification methods in determining the factors involved in *C. difficile* toxin production by different isolates.

## Figures and Tables

**Table 1:** Absolute quantification of TcdA and TcdB in small samples using MS^E^.

		Amounts of TcdA and TcdB studied (ng)				
Digested (ng)*	**63**	**125**	**250**	**500**	**1,000**	**2,000**
Expected (ng)**	**1.25**	**2.5**	**5**	**10**	**20**	**40**
TcdA						
Exp1***	0	0	3.08	8.7	16.46	31.7
Exp2	0	0	3.2	6.5	18.4	30.2
Exp3	0	0	3.4	6.3	16.3	32.7
**Average**	0	0	3.23	7.17	17.05	31.53
Stdev	0	0	0.16	1.33	1.17	1.26

TcdB						
Exp1***	0.84	1.1	2.05	7.9	14.1	36.05
Exp2	0.83	1.1	2.5	4.6	13.04	33.4
Exp3	0.83	1.2	3.4	4.6	13.7	26.4
**Average**	0.83	2.23	2.65	5.7	13.61	31.95
Stdev	0.01	0.06	0.69	1.91	0.54	4.99

Three samples each of purified toxin were serially diluted by a factor of 2 and digested with trypsin.

Each sample was analyzed three times using MSE. The numbers represent the amounts of toxin digested and the obtained values.

The minimum amounts of digested TcdA and TcdB in buffer that can be loaded on the nUPLC column and still detected were respectively 5 ng (1.6 *μ*g/mL) and 1.25 ng (0.43 *μ*g/mL).

*Values were estimated from the theoretical concentration based on values provided by the manufacturer.

**Amounts expected on column based on values provided by the manufacturer.

***Experimental values on column.

**Table 2:** Summary of the 24 most abundant *C. difficile* ATCC 43255 proteins identified and quantified in a commercial lyophilized culture filtrate.

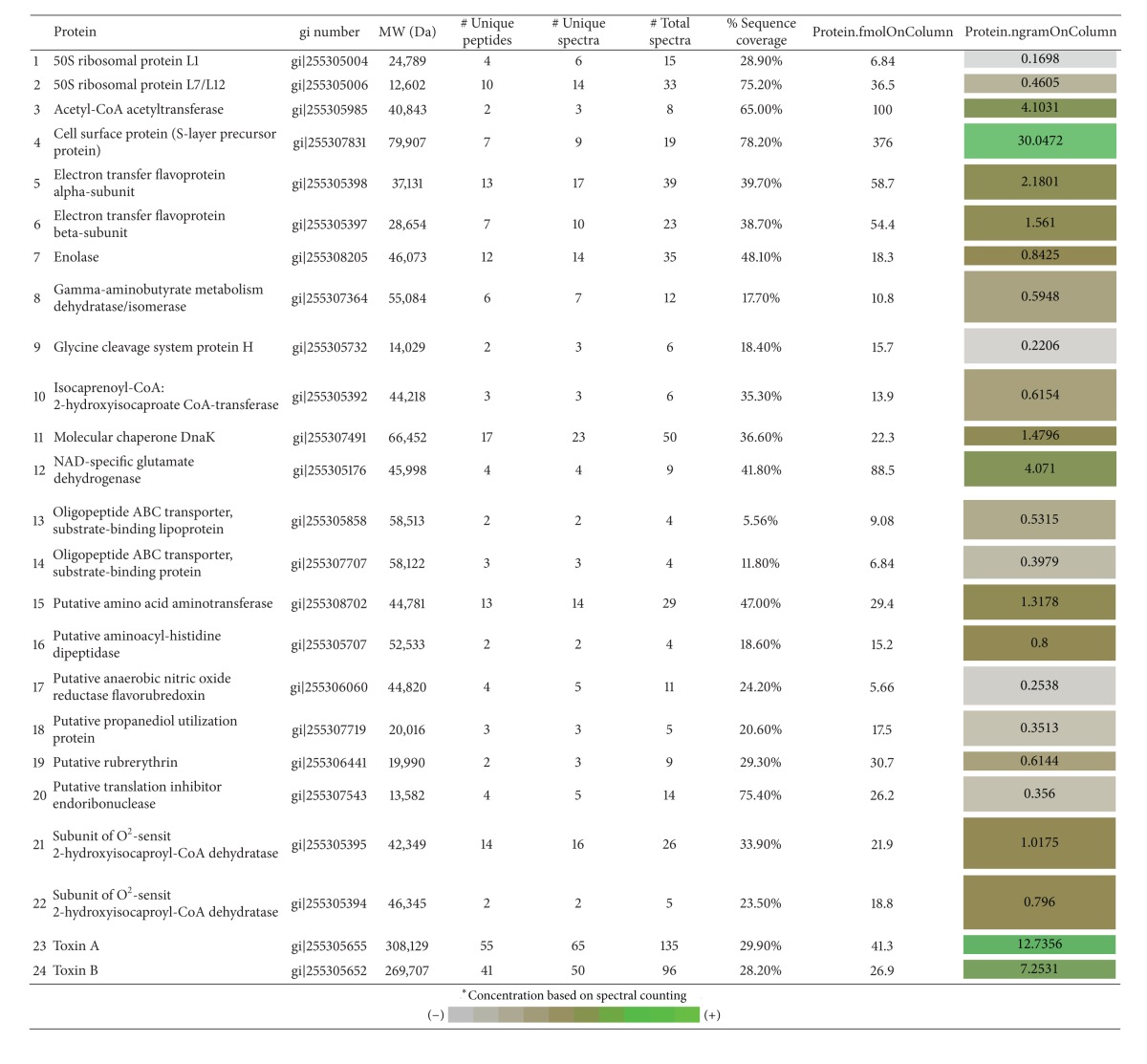

Proteins were identified using PLGS and Mascot search parameters and validated as greater than 95% peptide probability, min 2 peptides, 99% protein probability using Scaffold; FDR was zero. The colors in protein nanogram OnColumn are based on the estimated protein amounts.
